# Age-Related Changes in Insulin Resistance and Muscle Mass: Clinical Implications in Obese Older Adults

**DOI:** 10.3390/medicina60101648

**Published:** 2024-10-08

**Authors:** Ali A. Rizvi, Manfredi Rizzo

**Affiliations:** 1 Department of Medicine, Division of Endocrinology, Orlando VA Medical Center and University of Central Florida College of Medicine, Orlando, FL 32827, USA; 2 School of Medicine, Promise Department, University of Palermo, 90100 Palermo, Italy; manfredi.rizzo@unipa.it; 3 Ras Al Khaimah (RAK) Medical and Health Sciences University, Ras Al Khaimah 11172, United Arab Emirates

**Keywords:** obesity, older adults, sarcopenia, muscle mass, metabolic syndrome, diabetes

## Abstract

The older segment of the global population is increasing at a rapid pace. Advancements in public health and modern medicine lengthened life expectancy and reduced the burden of disease in communities worldwide. Concurrent with this demographic change is the rise in overweight people and obesity, which is evident in all age groups. There is also an aging-related reduction in muscle mass and function, or sarcopenia, that is exacerbated by sedentary lifestyle and poor nutrition. The coexistence of muscle loss and elevated body mass index, termed “sarcopenic obesity”, has particularly deleterious consequences in older individuals. Worsening insulin resistance and a proinflammatory state operate at the pathophysiologic level and lead to adverse health outcomes such as a proclivity to cardiovascular disease, type 2 diabetes, and even cognitive dysfunction. Although the concept of sarcopenic obesity as a disease construct is being increasingly recognized, a clearer understanding is warranted in order to define its components and health impact. Research is needed at the molecular-cellular level to tie together derangements in insulin action, cytokines, myokines, and endothelial dysfunction with clinical outcomes. Lifestyle modifications as well as targeted nonpharmacologic approaches, such as supplements and antioxidants, appear to have a promising role in reducing the chronic burden of this emerging disorder. Breakthroughs in drug therapies that retard or even reverse the underlying dynamics of sarcopenia and obesity in older persons are being actively explored.

## 1. Introduction

Obesity is a rapidly worsening global epidemic due to an increase in sedentary lifestyle and dietary changes. It is associated with multiple metabolic derangements leading to significant morbidity and mortality. It is projected that 85% of the adults in the United States will be overweight or obese by 2030 [1], a phenomenon that does not spare the older population [2]. In addition, older age is often associated with sarcopenia, a condition associated with progressive and generalized loss of muscle mass and a decline in muscle strength. The coexistence of obesity and sarcopenia is termed sarcopenic obesity (SO) [3,4]. The increasing prevalence of obesity became a compounding factor in ageing-related sarcopenia [5]. Older adults with SO are at higher risk of developing diabetes, hypertension, stroke, cardiovascular disease, and cognitive dysfunction, and have higher all-cause mortality than those who do not have SO [6,7]. In addition, the economic burden of SO is being recognized [8], highlighting the importance of its associations with cardiovascular diseases and mortality [9].

## 2. Age-Related Changes in Insulin Action

Insulin resistance (IR) is defined as the body’s relative inability to effectively utilize glucose, primarily in the liver, muscle, and adipose tissue, leading to progressive compensatory hyperinsulinemia. The clinical manifestations of IR are particularly augmented in the presence of obesity [10], leading to an elevated risk of prediabetes, diabetes, and cardiovascular disease. IR also underlies a clustering of risk factors for cardiometabolic atherothrombotic risk factors such as hyperglycemia, hypertension, dyslipidemia, and central obesity [11,12].

The hyperinsulinemic euglycemic clamp technique is widely acknowledged as the gold standard for assessing insulin sensitivity, particularly in research settings [13]. The commonest method of estimating insulin resistance is the Homeostatic Model Assessment of Insulin Resistance (HOMA-IR) [14], which is the product of fasting glucose (expressed as mg/dL) and fasting insulin (expressed as µU/mL), divided by a constant:

insulin resistance = (fasting glucose × fasting insulin)/405.

The concept of IR goes back to the days of Himsworth’s description [15], which was later developed and refined by Gerald Reaven at Stanford [16]. It is now recognized as the underlying metabolic abnormality in the clinical diagnosis of the metabolic syndrome [17]. Originally consisting of the four measurable parameters of central obesity, hypertension, hyperglycemia, and dyslipidemia, the definition was expanded to incorporate additional elements of liver dysfunction, hyperuricemia, and heightened inflammatory states [18]. Recent recommendations encourage clinicians to aggressively diagnose and manage the syndrome with lifestyle and pharmacologic therapies [19].

**Figure 1:**
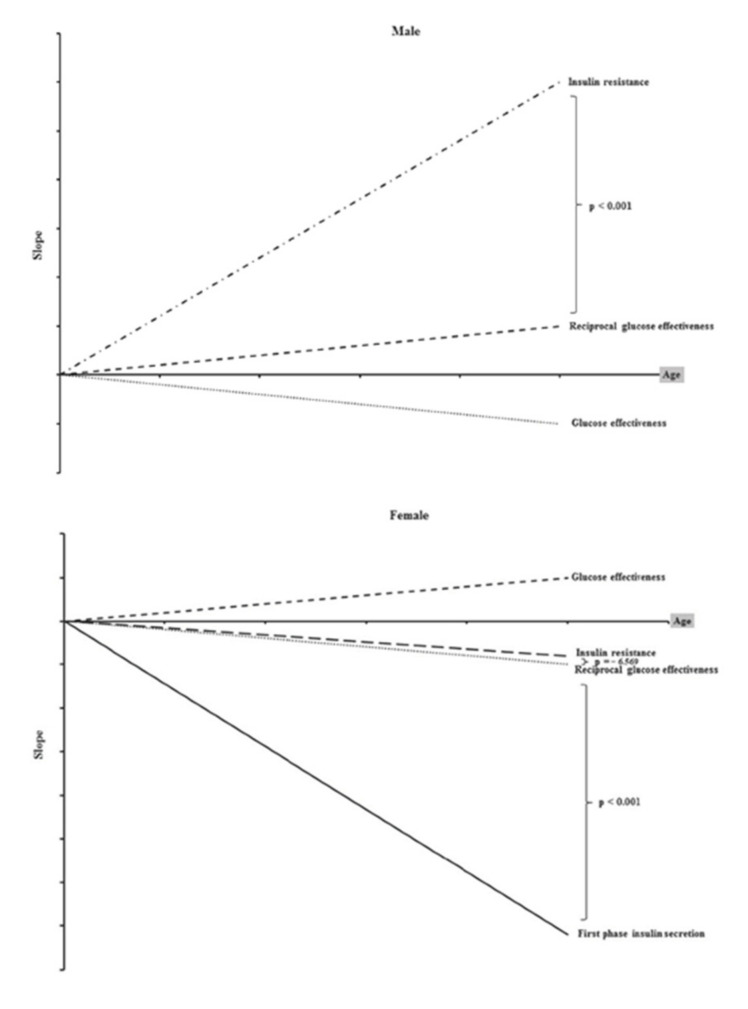
The relationships of insulin resistance, glucose effectiveness, and first-phase insulin secretion in men and women with age (from: Huang et al., 2023, available at https://www.mdpi.com/2075-4418/13/13/2158, accessed on 1 August 2024).

Advancing age is an important non-modifiable risk factor for worsening IR. The association between aging and reduced insulin sensitivity is not a predictably linear one, since it is influenced by factors such as adiposity, muscle mass, physical conditioning, chronic illnesses, and medication use [20]. In general, however, there is a gradual and inexorable decline in insulin effectiveness in the body with increasing age. Figure 1 shows the relationship between advancing age and insulin action.

The cellular and signaling mechanisms involved in changes in insulin action with age are not completely understood. The aging process alters the distribution of adipose tissue and adipogenesis, affects the inflammatory status and adipokine secretion, and enhances lipotoxicity. These age-dependent changes in fat are an important cause of insulin resistance and the subsequent development of type 2 diabetes (T2D) [21]. At the same time, sarcopenia sets in as an inexorable aspect of advancing age, which in turn affects the release of growth-promoting myokines and neurochemicals that could promote the resistance to insulin’s actions. The combined deleterious impact of these myoadipositic changes translates to increased frailty and reduced functional status [22].

## 3. Aging, Sarcopenia and Obesity

The decline in skeletal muscle mass and function is termed sarcopenia. The underlying mechanisms of increased sarcopenia with advancing age include marked alterations in muscle protein turnover and blunting of anabolic stimuli. Neural adaptations, alterations in neural components, and motor neuron excitability of the neuromuscular junction could also explain the changes. The impact of these changes on the older individual and public health systems can be significant. Loss of independence and increased frailty are often the result [22].

**Figure 2:**
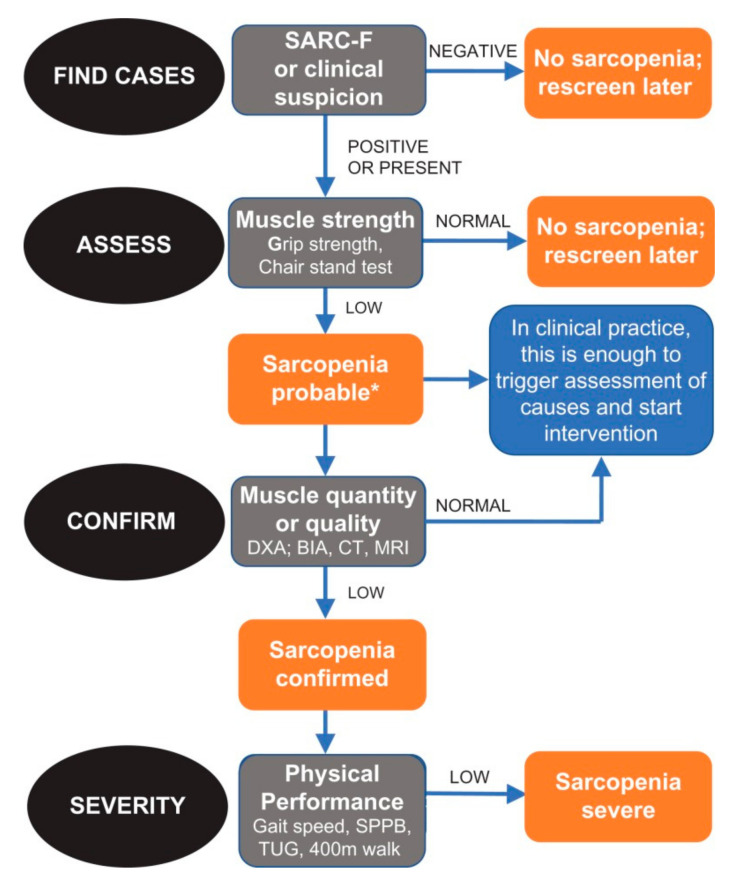
Sarcopenia: EWGSOP2 algorithm for case-finding, making a diagnosis and quantifying severity in practice. The steps of the pathway are represented as find-assess-confirm-severity or F-A-C-S. (from: Cruz-Jentoft et al., 2019, available at: https://www.ncbi.nlm.nih.gov/pmc/articles/PMC6322506/, accessed on 1 August 2024).

The methodology and objective assessment of sarcopenia evolved over the past two decades. The European Working Group on Sarcopenia in Older People (EWGSOP) developed sarcopenia cut-off points by age and gender [23]. It also suggested an algorithm for sarcopenia case finding in older individuals based on measurements of gait speed, grip strength, and muscle mass. Specific variables of muscle mass, muscle strength, and physical performance were used to a wide range of tools that can be used to measure the extent and severity of the condition. In 2019, the EWGSOP2 diagnostic algorithm was introduced encompassing sequential steps for case-finding, making a diagnosis, and quantifying severity in practice (Figure 2). This clinical tool also included cut-off points for sarcopenia based on examination of muscle strength, assessment of muscle mass, and evaluation of physical performance (Table 1). The presence of sarcopenia is categorised as probable, confirmed, and severe, respectively [24,25].

There is a paucity of knowledge about the interplay between obesity and sarcopenia in older adults. In addition to a high-calorie diet and physical inactivity, the presence of the metabolic syndrome and low-grade inflammation were implicated in increasing the likelihood of SO [26,27]. Conversely, SO may compound the risk of these clinical conditions much more than sarcopenia or obesity alone, and may be associated with significantly higher healthcare costs. Thus, the coexistence of obesity and sarcopenia creates a vicious trajectory promoting both muscle dysfunction and metabolic derangement [28]. Current management strategies for SO are focused on calorie restriction and increased physical exercise. Unfortunately, the feasibility of lifestyle interventions is often poor in SO patients due to physical limitations and the presence of chronic comorbid conditions [29]. We are in need of research that furthers our current understanding of how sarcopenia acts as a risk factor for early onset of type 2 diabetes.

The speculative mechanisms predisposing older obese adults to sarcopenia are listed in Table 2. It is unclear if SO is a necessary accompaniment of the elevated IR seen in people 65–85 years of age. Further, its relationship with glucometabolic and cardiovascular risk is yet to be fully explored. It is also unclear if glucose-intolerant, sarcopenic obese patients have higher plasma glucose, insulin, and hemoglobin A1c (HbA1c) readings compared to non-obese and non-sarcopenic subjects, or whether SO is associated with levels of inflammatory markers such as C-reactive protein (CRP), homocysteine, leptin, adiponectin, and interleukin 6 (IL-6) as well as with levels of myokines that promote muscle growth/maintenance [29,30].

Important parameters when quantifying SO include objective analyses of quality of life [31] and cognition [32]. A pooled analysis included three large datasets that examined the independent and combined associations of obesity and probable sarcopenia with all-cause mortality [33]. The risk of death increased for those having probable sarcopenia only (hazard ratio [HR]: 1.61, 95% confidence interval [CI]: 1.39–1.85) or probable sarcopenia with obesity (HR: 1.36, 95% CI: 1.13–1.64), but not for the obese-only group (HR: 0.92, 95% CI: 0.85–1.01), when compared to non-obese non-sarcopenic individuals. The authors concluded that maintaining muscle strength and identifying older adults at risk of sarcopenia would reduce premature mortality.

Chronic disorders such as T2D are postulated to accelerate the development of sarcopenia. T2D is thought to mediate this through increased insulin resistance, heightened inflammation, and accumulation of advanced glycation end-products [21]. Oxidative stress affects muscle mass and strength, protein metabolism, and vascular and mitochondrial dysfunction. Conversely, loss of muscle mass likely plays a role in the development of T2DM through the decreased production of myokines that adversely impacts metabolic function [34].

Because of its devastating impact on societies globally, the SARS-CoV-2 virus and the COVID pandemic deserve more than a passing mention when it comes to older sarcopenic individuals. A multitude of studies reported an increased risk of severe sarcopenia in COVID-19 patients during and after recovery. Acute sarcopenia in this context is often an under-recognised condition manifested by a rapid decline in muscle mass and function within six months of the insult, in this case, a severe COVID episode [35]. Lopez-Sampalo and colleagues prospectively evaluated body composition, muscle strength and the prevalence of sarcopenia, and the relationship between muscle strength with symptomatic and functional evolution in 106 older patients affected by COVID-19 [36]. They found a high percentage of sarcopenic patients at 3 months post infection (80%), especially among women and in those requiring hospitalization. However, at 12 months post infection, this percentage decreased considerably to approximately 55%, coinciding with both functional and symptomatic recovery, and the normalization of inflammatory parameters. The investigators concluded that older COVID-19 survivors could make a functional, clinical, and muscular recovery as long as aspects of nutrition and physical activity were given due attention. Finally, the association between sarcopenia and mortality in critically ill COVID-19 patients was examined in a review of 22 studies [37]. Of those, 17 studies reported a significant association, while 5 studies failed to show a link in severe COVID-19 illness. The authors recommended that the measurement of lean muscle mass be included in patients with severe COVID-19 as part of their prognostic risk assessment.

The combined mechanisms of chronic illness, malnutrition, and immobility, which cluster with aging, appear to be associated with the development of sarcopenia. The latter is often exacerbated by chronic comorbidities, including cardiovascular disease (CVD), chronic renal disease, and malignancies. Obesity is an overarching factor that promotes a negative impact on the overall health of the older person. The inanition of sarcopenia is also associated with faster progression of CVD, which undoubtedly leads to a higher risk of mortality among older adults [38]. It is imperative that the vicious and self-perpetuating cycle of musculoskeletal dysfunction and chronic disease be stemmed and reversed by proactively addressing the risk factors and instituting lifestyle changes in the older adults.

## 4. Association of Sarcopenic Obesity with Insulin Resistance

It is apparent that the increasing prevalence of older adults with diabetes became a major social burden. Diabetes, frailty, and cognitive dysfunction are closely related to the mechanisms of aging. Insulin resistance, arteriosclerosis, chronic inflammation, oxidative stress, and mitochondrial dysfunction may be common mechanisms shared by frailty and cognitive impairment. Hyperglycemia, hypoglycemia, obesity, vascular factors, physical inactivity, and malnutrition are important risk factors for cognitive impairment and frailty in older adults with diabetes.

Two major societal epidemics facing older adults are being overweight/obesity and sarcopenia. Although the prevalence and development of these conditions are studied in isolation of each other, the functional interplay between these two conditions is yet to be fully examined. It is unknown whether the coexistence of being overweight/obesity and sarcopenia (SO) is associated with elevated IR, thus predisposing one to increased cardiometabolic risk. Data point to a possible association between increasing weight (BMI) and waist-to-hip circumference ratio (WHR) and IR [39]. It is unknown if presence of sarcopenia, as measured by muscle mass and grip strength, has a substantial effect on this association. Plasma glucose and insulin levels would be expected to be higher in the sarcopenic obese individuals. The correlation of inflammatory markers and muscle function with body weight and degree of sarcopenia remains to be explored. Multiple variables could be assessed at a particular point in time, or longitudinally over an extended period.

The concept of SO and its association with the metabolic syndrome (MS) and cardiometabolic risk received close attention recently. In a cross-sectional analysis, SO was associated with MS and low-grade inflammation in Caucasian adults [40]. In total, 10.4% of participants in a Framingham cohort had SO when observed retrospectively at 10, 20, and 30 years [41]. They displayed a higher proportion of cardiometabolic risk factors such as hypertension, MS, and type 2 diabetes than the other three categories (*p* < 0.03). The investigators concluded that early recognition of sarcopenia provided an opportunity for interventions to reverse or delay the progression of muscle disorder, which may ultimately impact cardiovascular outcomes. Sarcopenia in the older population is associated with faster progression of cardiovascular disorders, a higher risk of mortality and falls, and reduced quality of life [38].

A cross-sectional analysis of the National Health and Nutrition Examination Survey III data shows an association of sarcopenia with IR in both non-obese (HOMA- IR ratio 1.39) and obese individuals (HOMA-IR ratio 1.16) and with glycemia in the latter [42]. IR leads to the dysregulation of key physiological processes that could be implicated in both metabolic and anabolic defects in skeletal muscle [43]. At a functional level, activities seem to be impacted roughly equally in older individuals, irrespective of the presence of obesity or sarcopenia (NHANES 1999–2004) [44]. However, these associations may be influenced by differences in IR among different body composition phenotypes. An older study suggested that the action of insulin does not play an important role in the development and maintenance of sarcopenia in healthy, non-obese postmenopausal women [45]. In the Concord Health and Ageing in Men Project, SO did not appear to confer greater risk for incident MS or IR than obesity alone in community-dwelling older men [46].

**Figure 3:**
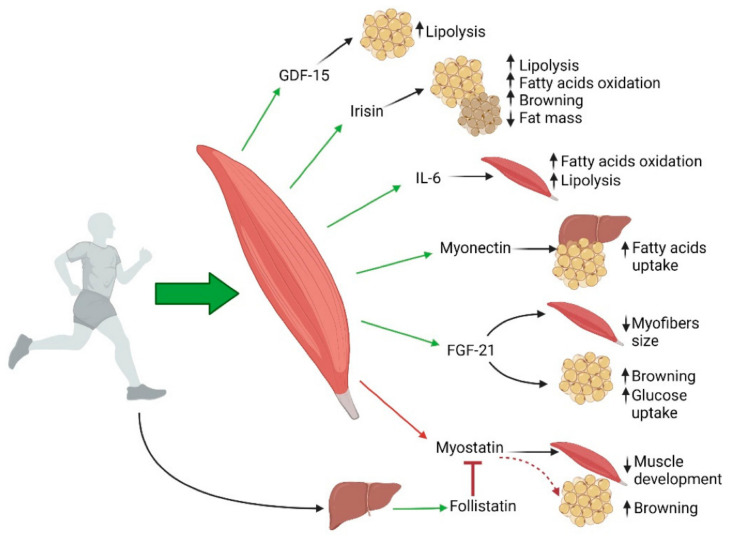
Diagrammatic depiction of myokines exerting paracrine and autocrine effects because of contraction of skeletal muscles. Release of stimulatory cytokines and the inhibitory mediator are shown by green and red arrows, respectively, while black arrows indicate downstream effects. GDF-15 = growth differentiation factor-15, IL-6 = interleukin-6, and FGF-21 = fibroblast growth factor-21. (From: Feraco et al., 2021, available at https://www.ncbi.nlm.nih.gov/pmc/articles/PMC8430804/, accessed on 1 August 2024).

The inverse association of relative muscle mass with insulin resistance and prediabetes was noted in population-based, epidemiologic data [47]. Similarly, the interplay between muscle mass decline, obesity, and type 2 diabetes was highlighted [48]. Skeletal muscle is being increasingly recognized as an endocrine organ, synthesizing and secreting several cytokines and peptides [49]. These “myokines” are released in response to muscular activity, facilitating an autocrine, paracrine, and endocrine hormonal crosstalk with other organs (Figure 3). Sedentary lifestyle impairs their secretion, thus predisposing to a variety of chronic illnesses. Fat accumulation in the muscle, with or without the presence of obesity, may explain some of the functional and metabolic defects shown in the frail, sarcopenic population [50]. The loss of muscle mass is often seen in conjunction with that of bone, a phenomenon sometimes termed “osteosarcopenia” [51]. The biomechanical, cellular, paracrine, endocrine, neuronal, and nutritional perturbations in muscle–bone interactions may contribute to the pathophysiology of this condition.

**Figure 4:**
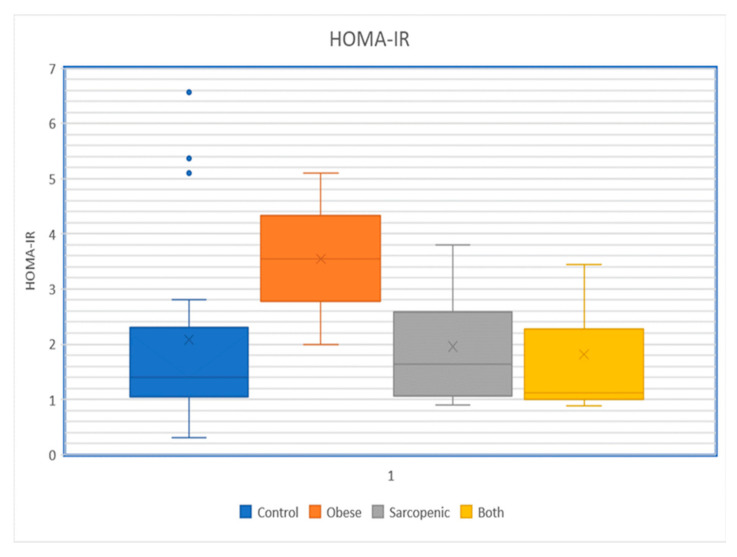
HOMA-IR Index in the control (nonobese–nonsarcopenic), obese–nonsarcopenic, nonobese–sarcopenic, and obese–sarcopenic study groups: mean HOMA-IR values were 2.35, 4.38, 2.60, and 2.22, respectively, (from Rizvi et al., 2024, available at: https://doi.org/10.2337/db24-158-OR).

Preliminary observations from our group indicate some interesting findings regarding sarcopenia in overweight/obese individuals compared to those of normal weight [52]. We investigated whether IR was significantly associated with being overweight/obesity, central fat deposition, and muscle mass and/or function in 77 adults (43 females, 34 males, age range 65–80 years, mean age 71 years). The following anthropometric and laboratory data were obtained: body mass index (BMI, as a determinant of overweight/obesity); waist circumference (WC, abdominal obesity); thigh circumference and grip strength (measures of muscle mass and sarcopenia); fasting glucose and plasma insulin levels (for calculation of HOMA-IR); and hemoglobin A1c. Thirty-two of the participants (42%) were normal weight, and 45 (58%) were overweight/obese. Fifteen individuals (19%) were classified as sarcopenic, and the prevalence was similar in normal weight vs. overweight/obese groups (19% vs. 20%). Selected mean parameters were: BMI 28.05, WC 113.8 cm, and HOMA-IR index 1.73 (normal < 1). Analysis of the data shows a statistically significant positive association of HOMA-IR with BMI and WC (*p* < 0.05), but not with sarcopenia (Figure 4). The WC correlated with glucose and insulin levels in both sexes (*p* < 0.05) and with A1c in females only. Findings from our study thus far do not support a higher prevalence of sarcopenia in overweight/obese individuals than in those of normal weight. In both sexes, IR was significantly associated with overweight/obesity and central fat deposition, but not with sarcopenia. Interventions primarily targeting weight loss, with or without enhanced muscle conditioning, would be expected to be most effective in ameliorating IR and reducing cardiovascular and metabolic risk in older adults. In other words, sarcopenia per se is not likely to be more prevalent in older individuals with greater degrees of IR. The latter appears to correlate more with obesity and its related comorbidities and with glucose intolerance only in females. These findings challenge the concept of “sarcopenic obesity” as a single pathophysiologic and clinical construct.

## 5. Changes in Sarcopenic Obesity and Functional Capacity with Aging

The progression of sarcopenia with aging reflects a progressive blunting of anabolic processes, an increased catabolic state, and diminished muscle regeneration capacity [53]. Muscle force and power in older individuals decline more than muscle dimensions. Older muscle is therefore intrinsically weak, and SO among the elderly parallels the loss of muscle mass and increased relative fat. A multiplicity of factors, including nutritional, hormonal, neural, and immunological, coupled with the level of physical activity, contribute to sarcopenia of aging exacerbated by concomitant obesity. The consensus appears to be that obese individuals, regardless of age, have a greater absolute maximum muscle strength compared to non-obese persons. This could reflect increased adiposity functioning as a negative factor on the musculature, thus affecting muscle size and strength [54]. SO can lead to functional limitations in muscle performance and increased likelihood of developing a disability stemming from loss of mobility and postural balance. Animal studies showed that older rats displayed a reduced ability to oxidize fatty acids within the muscle cell, rendering them prone to ectopic muscle lipid accumulation and decreased muscle protein formation [55]. This age-related metabolic alteration is a potential therapeutic target in efforts to counter sarcopenic obesity. Nevertheless, the progressive impact of obesity upon skeletal muscle size, structure, and function with aging remains to be fully explained.

## 6. Clinical Implications of Sarcopenic Obesity in Older Adults

Lifestyle interventions incorporating both diet-induced weight loss and regular exercise appear to be the optimal treatment for sarcopenic obesity [56,57]. A meta-analysis showed that a low-calorie high-protein diet and aerobic exercise decreased body weight and fat mass but did not improve physical performance [58]. Resistance exercise improved body composition, physical performance, and grip strength in individuals with SO, emphasizing the importance of preserving skeletal muscle mass while reducing fat mass [59,60,61]. Overall dietary quality, appropriate intake of calories and protein, supplementation with specific ergogenic or branched-chain amino acids, and consumption of antioxidant nutrients, vegetables, fruits, and protein, may be beneficial in warding off SO [62,63]. Essential amino acid supplementation appears to be effective for enhancing muscle mass and strength in the elderly, but its role in reducing fat mass and managing SO is less certain [64]. Since oxidative stress could be a mechanism underlying sarcopenic obesity, a protective role of antioxidant flavonoids appears promising [65].

A supervised, moderate caloric restriction coupled with regular aerobic and resistance exercise in obese older adults may reduce insulin resistance, metabolic complications, and disabilities in older adults without exacerbating lean mass and bone mineral density loss [66]. These mechanisms, acting through dietary protein and physical activity modulation, are proposed to contribute to prevention of chronic diseases such as diabetes [67]. Maintaining the appropriate frequency and intensity of resistance training can help prevent muscle atrophy and effectively reduce musculoskeletal inflammation. Physical activity also has the ability to slow β cell dysfunction and promote protein synthesis in the elderly [68]. The potential benefits of combined non-pharmacological approaches including food composition, protein intake, exercise, dietary supplements, changing the gut microbiome, and rehabilitation therapies were advocated [69]. Sedentary older (>60 years of age) residents of long-term care facilities benefitted from resistance exercise through improvements in muscle strength, body composition, and physical capacity [70]. The effect of whole-body electromyostimulation (WB-EMS) as an emerging modality was evaluated in 75 community-dwelling women >70 years of age with SO [71]. The intervention demonstrated an increase in muscle mass and functional capacity; however, the effect on body fat in these older women was not significant. The addition of protein-enriched supplements did not add to the benefits of WB-EMS.

Bariatric surgery demonstrated effective weight loss and improvement in comorbidities with a favorable safety profile in individuals with SO compared to the non-sarcopenic group [72]. However, its role in improving activities of daily living and functional autonomy remains unproven. In the oldest (≥80 years), as in frail individuals, it seems reasonable to abstain from recommending significant weight loss.

The clinical implications of the current knowledge in this field are twofold. Interventions primarily targeting weight loss, with or without enhanced physical activity, would be expected to be most effective in ameliorating IR and reduce cardiovascular and metabolic risk in older adults; and concurrently, the mutable determinants of ageing-related sarcopenia, namely suboptimal nutrition and physical deconditioning, need to be simultaneously addressed for optimizing quality of life in older persons. With the rapid increase in ageing in societies globally, there is a need to bridge the gap between research and clinical practice in this field through improvement and utilization of currently accepted diagnostic tools and criteria.

## Figures and Tables

**Table 1:** EWGSOP2 Sarcopenia Cut-Off Points (modified from: Cruz-Jentoft et al., 2019, available at https://www.ncbi.nlm.nih.gov/pmc/articles/PMC6322506/, accessed on 1 August 2024).

Test	Men	Women
Grip strength using calibrated handheld dynamometer	<27 kg	<16 kg
Chair stand for leg muscle strength	>15 s for five rises	
Appendicular skeletal muscle mass (ASM) (with MRI, CT, or DEXA)	<20 kg	<15 kg
ASM/height^2^ (to adjust for body size)	<7.0 kg/m^2^	<5.5 kg/m^2^
Gait speed	≤0.8 m/s	
Short physical performance battery (SPPB)	≤8-point score	

**Table 2:** Speculative Mechanisms Predisposing to Sarcopenia in Older Adults with Obesity.

Elevated insulin resistance (reduced insulin action and compensatory hyperinsulinemia)
Role of inflammatory markers (e.g., C-reactive protein, homocysteine, leptin, adiponectin, and interleukin 6)
Reduced levels of myokines (e.g., myostatin, irisin, and fibroblast growth factor-21)

## Data Availability

Not applicable.
